# Myocardial Ischemia with Cannabinoid Use in an Adolescent

**DOI:** 10.7759/cureus.1899

**Published:** 2017-11-30

**Authors:** Jeet J Mehta, Arjun K Mahendran, Ravi K Bajaj, Arpan R Doshi

**Affiliations:** 1 Internal Medicine/pediatrics, University of Kansas School of Medicine - Wichita; 2 Pediatrics, University of Kansas School of Medicine - Wichita; 3 Heartland Cardiology, University of Kansas School of Medicine - Wichita; 4 Pediatric Cardiology, Children's Mercy Hospitals & Clinics, University of Kansas School of Medicine - Wichita

**Keywords:** pediatric cardiology, cannabinoids, adolescent, acute coronary syndrome, synthetic, marijuana, marijuana legalization, myocardial ischemia

## Abstract

A 16-year-old male presented to the emergency department with chest pain after smoking a synthetic cannabinoid from a vape pen. He had rising troponin I levels, and his exercise stress echocardiogram showed distal apical and septal hypokinesis that resolved at six-month follow-up. This case report raises concern about cardiac ischemia related to synthetic cannabinoid abuse in the pediatric population in the current era of cannabis legalization.

## Introduction

In the current era, the reported prevalence of synthetic cannabinoid use ranges between 6.5% and 12.6% in adolescents and adults in the United States and United Kingdom [1]. The prevalence of use of both synthetic and plant-based cannabis is expected to rise with the movement of the legalization of cannabis. Acute transient side effects of marijuana are well known to medical professionals, but few cases are reported with cardiac ischemia in the pediatric population [2]. We report a case of a teenager presenting with myocardial ischemia after synthetic cannabinoid use.

## Case presentation

**Figure 1:**
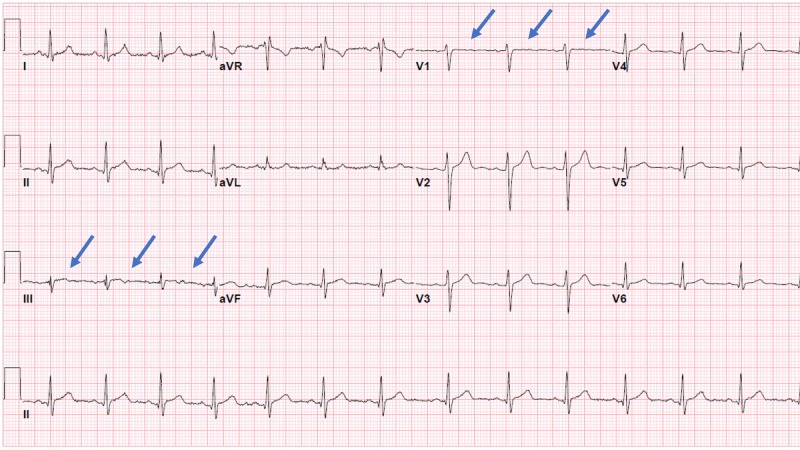
ECG upon admission Admission ECG showing non-specific ST segment changes in inferior and septal leads without ST-segment elevation ECG: electrocardiogram

A 16-year-old obese Caucasian male presented to the emergency department with the sudden onset of left-sided chest pain. His physical exam was unremarkable, except for an elevated blood pressure of 142/76 mmHg and a BMI of 39 kg/m2 (>99th percentile). His troponin I was elevated at 1.63 ng/ml at his initial presentation. Renal function was normal. His initial ECG (electrocardiogram) showed a sinus rhythm with the presence of non-specific ST-segment changes in the inferior and septal leads without ST-segment elevation as seen in Figure 1.

**Figure 2:**
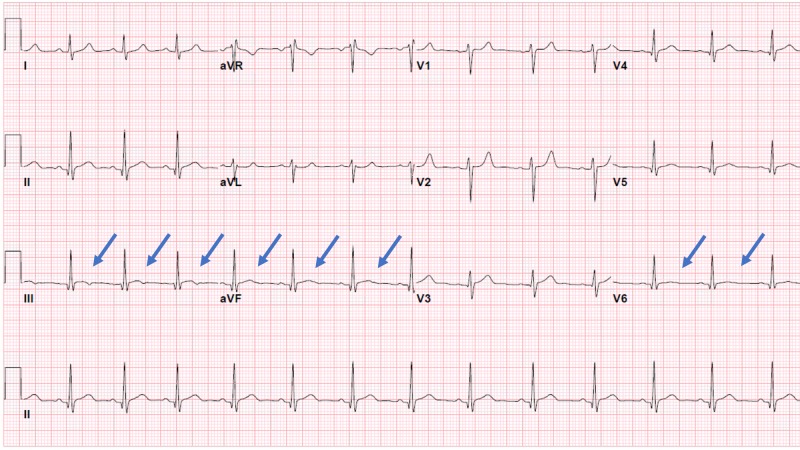
ECG at discharge Discharge ECG showing normal sinus rhythm with nonspecific ST-T-wave changes in inferior and lateral leads ECG: electrocardiogram

At this point, the patient was transferred to a regional children’s hospital. Family history was positive for hypertension in the mother at 43 years of age, coronary artery disease in father before age of 50 years, and heart failure in extended family members. A fasting lipid panel revealed elevated triglycerides at 181 mg/dL and a low HDL (high-density lipoprotein) at 23 mg/dL with a normal hemoglobin A1c of 5.1%. His CRP (C-reactive protein) and ESR (erythrocyte sedimentation rate) levels were normal on admission. Repeat troponin I at a six-hour interval from the presentation showed further elevation to 3.32 ng/ml. The patient initially declined use of illicit substances but later admitted to smoking a “vape pen” for the first time prior to the onset of chest pain. His drugs of abuse urine screen was positive for cannabinoids. A baseline transthoracic echocardiogram showed a structurally normal heart with normal biventricular function. The next day, his troponin I level trended down to 2.64 ng/mL; however, there was a rise in the troponin I level to 3.19 ng/mL on Day 3. There was a strong suspicion of synthetic cannabinoid use-related coronary artery vasospasm as the cause of the elevated troponin I levels. An exercise stress echocardiogram was obtained to further evaluate the elevation of troponin I. The stress test showed evidence of distal septal and apical wall hypokinesis, indicating myocardial ischemia in the left anterior descending coronary artery distribution. On the day of discharge, his troponin I was down to 0.69 ng/ml, and an ECG showed normal sinus rhythm with nonspecific ST-T changes in the inferolateral leads as seen in Figure 2.

The patient was discharged home on dual-antiplatelet therapy with clopidogrel and aspirin, a statin, metoprolol, and nitroglycerin as-needed with the stress echocardiography findings and patient's susceptibility of coronary artery disease with risk factors of a strong family history, obesity, and dyslipidemia. Extensive counseling and resources were provided to the patient regarding substance abuse. At his two-month follow-up, the patient remained asymptomatic and he was continued on the same medical therapy. A subsequent exercise stress test was negative for regional wall motion abnormalities and ischemia at his six-month follow-up, at which point the clopidogrel and metoprolol were discontinued; however, he was continued on aspirin, the statin, and as-needed nitroglycerin.

## Discussion

The use of synthetic cannabinoids is on the rise in the United States, especially in the adolescent population. Typical acute side effects of synthetic cannabinoid are tachycardia, hypertension, lethargy, nausea, irritability, chest pain, hallucination, and confusion [2-3]. Cannabinoids are known to increase myocardial oxygen demand and are also hypothesized to cause coronary vasospasm, resulting in ischemia [4]. There are reports of significant cardiovascular effects, including myocardial infarction, in the adult population [5]. Data regarding such cardiovascular effects in the pediatric population is scarce [6]. Further data regarding diagnostic testing and treatment of synthetic cannabinoid-related cardiovascular side effects is also needed. This case report sheds light on a potentially life-threatening side effect of synthetic cannabinoid use in an individual susceptible to coronary artery disease.

## Conclusions

Our patient presented with chest pain and elevated troponin I suggesting myocardial ischemia after a presumed synthetic cannabinoid inhalation from a "vape pen". His stress echocardiogram showed regional wall motion abnormalities in the distal left anterior descending coronary artery distribution, which had normalized by the time of the six-month follow-up. His CRP and ESR were normal on admission, making the diagnosis of myocarditis unlikely. We suspect our patient had coronary vasospasm secondary to synthetic cannabinoid use causing transient myocardial ischemia and regional wall motion changes. This case report serves as an alert to medical providers to be mindful of myocardial ischemia after synthetic cannabinoid use in the current setting of increased prevalence of cannabinoid use.
